# Continuous-wave phase-matched molecular optical modulator

**DOI:** 10.1038/srep20908

**Published:** 2016-02-18

**Authors:** Shin-ichi Zaitsu, Hirotomo Izaki, Takao Tsuchiya, Totaro Imasaka

**Affiliations:** 1 Department of Applied Chemistry, Graduate School of Engineering, Kyushu University, 744 Motooka, Nishi-ku, Fukuoka 819-0395, Japan; 2 PRESTO, Japan Science and Technology Agency (JST), 4-1-8 Honcho, Kawaguchi, Saitama 332-0012, Japan; 3 Division of Optoelectronics and Photonics, Center for Future Chemistry, Kyushu University, 744 Motooka, Nishi-ku, Fukuoka 819-0395, Japan

## Abstract

In optical modulation, the highest available modulation rate is basically limited to the GHz frequency range at best. This is because optical modulation is often performed using electro-optic or acousto-optic effects that require application of an external signal to solid-state nonlinear optical materials. Here we describe optical modulation of continuous-wave radiation at frequencies exceeding 10 THz based on ultrafast variation of molecule polarizability arising from coherent molecular motion. The optical modulation efficiency is extensively enhanced by fulfilling phase-matching conditions with the help of dispersion control of the optical cavity, generating sidebands with a highest ratio of 7.3 × 10^−3^. These results will pave the way for development of versatile optical modulation-based techniques in a wide range of research fields in optical sciences, such as mode-locked lasers operating in the THz range.

Light manipulation is essential for the use of light in a wide range of scientific research fields. Optical modulation techniques are used to modify light characteristics in a periodic or quasi-periodic manner and play a principal role in optical methods such as spectroscopy and microscopy, optical communications, and other applications^1^,^2^. Optical modulation is often performed using solid-state nonlinear optical materials that exhibit electro-optic or acousto-optic effects. The highest available modulation rate in these approaches is basically limited by the frequency of the AC voltage (~GHz) or acoustic wave (~MHz) applied to the nonlinear optical materials. To expand the optical modulation range, various strategies, including the ultrafast Kerr effect in silicon-polymer hybrid materials^3^ and re-collision of excitons in a quantum well under THz-wave irradiation^4^, have been reported, and have achieved optical modulation of continuous-wave (cw) light at frequencies in the THz range.

C. H. Townes, one of the inventors of the laser, pointed out in 1963^5^ that the Stokes and anti-Stokes emissions in the coherent Raman process, i.e. the generation of Raman sidebands, can be explained by optical modulation based on the periodic variation of polarizability that arises from coherently excited molecular motion. Townes’s idea could be called “molecular optical modulation (MOM)”, and it leads to the concept of an optical modulator operating at THz frequencies (see Fig. 1a), i.e. a “molecular optical modulator”, because the frequencies of the required molecular motion are generally in the THz (10^12^ − 10^14^ Hz) range. However, the realization of such an ultrafast optical modulator has taken a long time from the birth of the original concept. This is mainly because of the difficulty involved in fulfilling the phase-matching condition that determines the optical modulation process efficiency for a Raman shift frequency when approaching a carrier wave frequency. As illustrated by the spectrum and the energy diagram shown in Fig. 1b,c, respectively, sideband generation on the higher and lower sides of the centre frequency during optical modulation is equivalent to degenerate four-wave mixing (DFWM). The phase mismatch between the fundamental beam and the sidebands in this DFWM process, Δ*k*, is approximated by the following equation when the group delay dispersion (GDD) of the molecular medium is denoted by *β* and any even-order dispersion terms higher than the fourth order are ignored^6^:


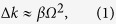


where *Ω* is the Raman shift frequency, which corresponds to the operating frequency of the molecular optical modulator. The inherent parameter, *β*, is determined by the medium, and *β* > 0 generally applies in the optical region. When *Ω* is in the GHz (10^9^ − 10^10^ Hz) region, then Δ*k* is negligibly small; however, it cannot be ignored for *Ω* values of more than 10^12^ Hz, because it degrades the sideband generation efficiency dramatically. This difficulty has been overcome by using a quantum interference effect, electromagnetically induced transparency (EIT)^7^, which is controlled by an intense pump “pulse” laser. CW operation of a molecular optical modulator, which is expected to be applied in a wide range of fields, requires steady-state excitation of molecular coherence. This is achieved, in principle, by cw excitation of molecular coherence.

To turn the MOM concept into practical applications, a scheme with higher efficiency at moderate pump powers is desirable. D. D. Yavuz proposed the theory of a frequency mixer operating in the THz region for cw radiation using coherent Raman-active molecules in a high-finesse optical cavity excited by a cw laser^8^. The optical modulation of a cw laser at the motional frequency of molecules was later observed experimentally using this approach^9^. However, the pump power level used (~20 W) in this approach was insufficient to stimulate EIT, resulting in an efficiency (the ratio of the intensity of the Raman sidebands to that of the fundamental beam included in the output beam) of 2 × 10^−6^. An alternative way to improve the MOM efficiency is to extend the light-molecule interaction length under phase-matched conditions all the way through the process. According to Eq. (1), the phase mismatch, Δ*k*, is determined by the GDD of a Raman-active medium, *β*. This indicates that the elimination of *β* leads to the maximum optical modulation efficiency under the phase-matching condition Δ*k* = 0. Unfortunately, *β* generally has a nonzero value and a positive correlation to the concentration of the medium in the optical region, hindering fulfilment of the phase-matching condition for gaseous isotropic media. To solve this problem at a moderate pump power, we consider MOM arising from molecules contained in an optical cavity consisting of multiple highly reflective mirrors with designed dispersion properties. In this case, the GDD effect on an intracavity beam is caused by two factors: (1) the medium contained in the cavity, and (2) the cavity mirrors, which have dispersive properties caused by variation of the Bragg wavelength^10^. Let *β*_medium_ be the GDD produced by the medium for a round trip pass through the optical cavity and let the sum of the GDDs for one bounce off all the mirrors that comprise the cavity be *β*_mirror_. The total GDD related to the MOM process is then expressed as:


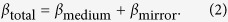


When MOM is performed in a dispersion-designed optical cavity, *β*_mirror_ and *β*_medium_ must be taken into account to evaluate the phase mismatch. Fortunately, broadband highly reflective mirrors with “negative” GDD values (*β*_mirror_ < 0) are available following recent advances in ultrafast laser technology^11^. When the optical cavity for MOM is constructed using negatively dispersive mirrors with appropriate negative GDD values, the “positive” value of *β*_medium_ can be cancelled out, i.e. *β*_medium_ + *β*_mirror_ = 0. This “dispersion-compensated optical cavity” allows us to satisfy the phase-matching condition in a “quasi” manner throughout the intracavity MOM process, and thus offers the potential to realize MOM with higher efficiency under phase-matched conditions (see Fig. 2a).

From the frequency domain viewpoint, this idea can be understood as an overlap between the sideband frequencies, *ω*_1_ and *ω*_−1_, and the longitudinal modes of the optical cavity (see Fig. 2b,c). The frequency spacing between the adjacent longitudinal modes of the optical cavity (free spectral range) is expressed by the reciprocal of the group delay for one round trip of the beam in the optical cavity^12^: *δΩ* = 1/*τ*. In a medium-filled optical cavity, *τ* is determined by two factors:


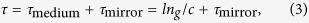


where *τ*_medium_ is the group delay determined by the group refractive index of the intracavity medium *n*_*g*_ the optical cavity round-trip length *l*, and the speed of light in a vacuum, *c. τ*_mirror_ is a group delay given by the sum of contributions of bounces from all the cavity mirrors. In the simplest scenario, when *τ* has no frequency dependence, *δΩ* is constant at all frequencies. In reality, however, *n*_*g*_ and *τ*_mirror_ have non-negligible frequency dependences, leading to non-constant intervals between the longitudinal modes. In contrast, the energy conservation law in sideband generation requires equally-spaced intervals between the fundamental frequency and the sideband frequencies, i.e. *ω*_1_ = *ω*_0_ + *Ω* and *ω*_−1_ = *ω*_0_ − *Ω*. This causes frequency mismatches, Δ*Ω*_1_ and Δ*Ω*_2_, at each sideband (see Fig. 2b). Note that Δ*Ω*_1_ and Δ*Ω*_2_ are not always non-zero when *τ*_mirror_ has an appropriate negative value, even though *τ*_medium_ has a positive value. Because *n*_*g*_ can be controlled by varying the concentration of the intracavity gaseous medium, we can determine an optimized condition where both Δ*Ω*_1_ and Δ*Ω*_2_ are zero (see Fig. 2c). Under this condition, *ω*_1_ and *ω*_−1_ are matched with the longitudinal modes, and the sidebands that arise from the MOM process are allowed to interact with the molecular coherence over a long intracavity distance determined by the cavity finesse. This means that the phase-matching condition for intracavity MOM is satisfied under the condition where *β*_total_ = 0, as described in the preceding paragraph. From a qualitative perspective, this indicates that the phase slip between the sidebands caused by a half-round trip in the medium-filled optical cavity is compensated by a single bounce on a cavity mirror with negative dispersion (see Fig. 2a, bottom right). In this way, compensation of the optical cavity dispersion helps to maintain the phase relationship between the fundamental beam and the sidebands, leading to so-called “quasi”-phase-matched optical modulation at operating frequencies in the THz region.

We should mention here an important technique that is based on the nonlinear polarizability caused by Raman polarization, “Raman-induced Kerr effect (RIKE)” reported in the early stage of Raman spectroscopy^13^. In a typical RIKE spectroscopy experiment, a frequency difference between the pump and probe beam is tuned to match the Raman shift frequency of a target molecule and a low background Raman signal in the Stokes beam can be obtained with a high scattering efficiency^14^. The effect of the phase-matching in the Raman process is ignored in this scheme because it relies on stimulated Raman scattering. Oh the other hand, in this work, the nonlinear Raman polarization affects on a frequency-tunable probe beam, and four-wave mixing contributes to the generation of sidebands, an anti-Stokes component as well as a Stokes component. Unlike the RIKE spectroscopy, the fulfillment of the phase-matching in our approach is an essential factor, which is achieved by an appropriate control of the cavity dispersion.

## Results

### Pump and probe of molecular coherence

Figure 3 shows a simplified schematic of the experimental setup used to demonstrate MOM in this study, including the light sources, a gas-filled chamber containing the optical cavity, the system used to control the optical cavity and the optics that couple the pump and probe beams to the optical cavity. In principle, coherent molecular motion can be resonantly excited using a beam that includes two frequencies (*ω*_P1_ and *ω*_P2_) with a separation that corresponds to the frequency of molecular motion. In this experiment, *ortho*-H_2_, which has a 17.6 THz rotational motion frequency, is used as a modulation medium and the coherence is created through a two-photon transition, *S*_1_(0). This means that the operating frequency of this MOM is 17.6 THz. The gaseous hydrogen is used to fill the chamber that encloses a Fabry–Perot-type optical cavity composed of negative dispersion mirrors. A single-frequency cw laser beam from a Ti:sapphire laser operating at a frequency of *ω*_P1_ is coupled to the hydrogen-filled optical cavity, and this beam and a Stokes beam, which is generated by intracavity stimulated Raman scattering (SRS)^15^ at a frequency of *ω*_P2_, are used as the pump beams. The estimated intensities of the cavity-enhanced pump beams (*ω*_P1_ and *ω*_P2_) are 3.8 × 10^5^ W/cm^2^ and 3.7 × 10^5^ W/cm^2^, respectively. This leads to the coherence, *ρ*, of the molecular rotation, which is calculated to be *ρ* = 3.7 × 10^−5^ at the maximum (see Methods for details). Further details of the experimental setup are described in the supplementary information.

### Estimation of the phase-matching conditions

**Figure 2:**
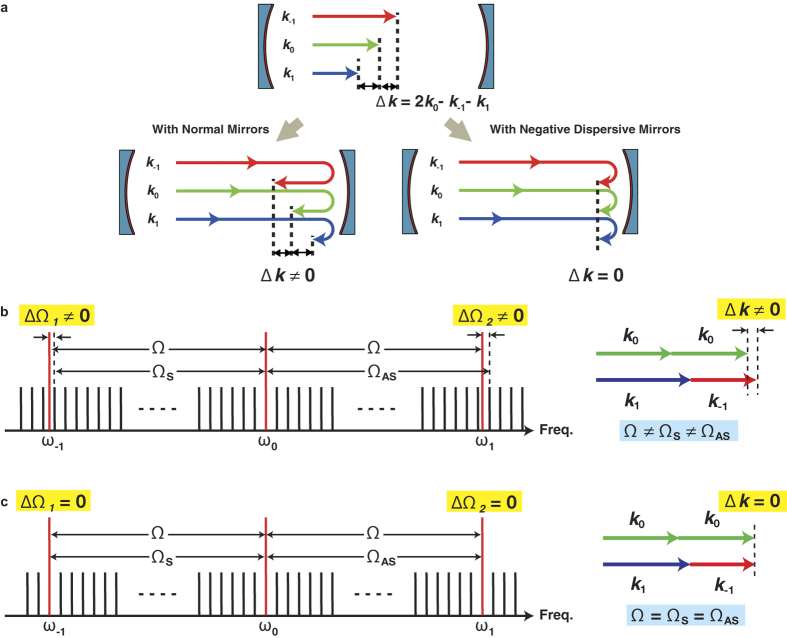
Concept of phase matching in intracavity optical modulation. (**a**) Comparison between the behaviour of wave vectors that bounce on a positively dispersive cavity mirror (bottom left) and a negatively dispersive cavity mirror (bottom right). The phase mismatch, Δ*k*, which is caused by the beam passing through an intracavity medium, can be compensated by bouncing the beam off a negatively-dispersive cavity mirror. (**b**) Longitudinal modes of the optical cavity and the wavelengths related to the molecular optical modulation (MOM) process. Top: for a non-dispersion-compensated optical cavity, where neither Δ*k* nor the frequency mismatch, Δ*Ω*, are zero. Bottom: for a dispersion-compensated optical cavity, where both Δ*k* and Δ*Ω* are zero.

**Figure 3:**
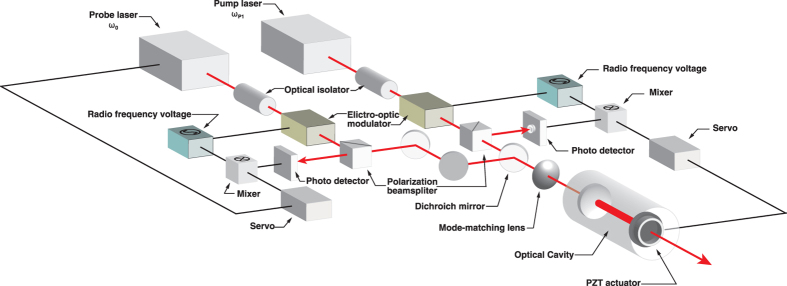
Schematic of the experimental setup. Two single-frequency tunable Ti:sapphire lasers are used to provide a pump beam and a probe beam. These beams are combined with a dichroic mirror and coupled to an optical cavity filled with pressurized hydrogen. The resonance condition of the optical cavity is stabilized using two independent Pound–Drever–Hall systems. See the supplementary information for details.

**Figure 4:**
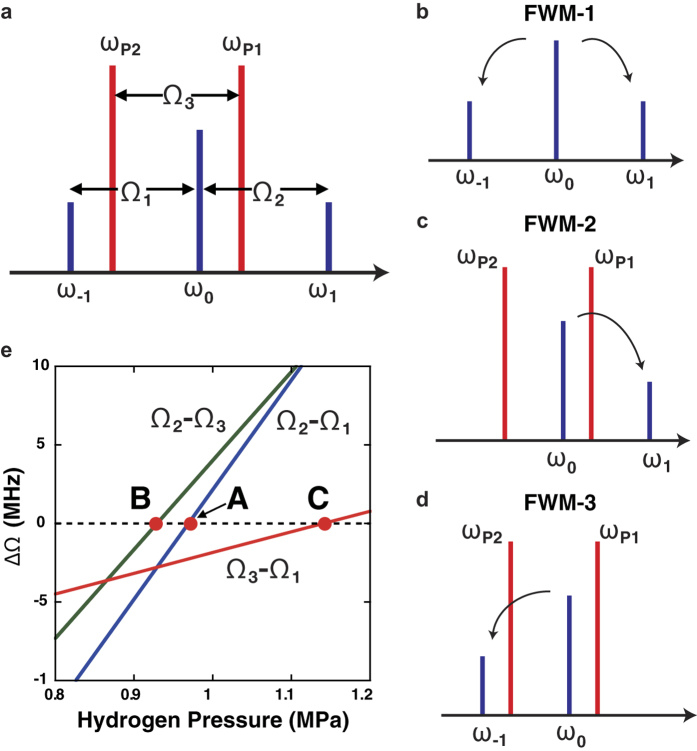
Three types of FWM involved in the intracavity MOM process. (**a**) Five longitudinal modes related to the MOM process. (**b**) FWM-1: degenerate four-wave mixing (FWM). Both *ω*_1_ and *ω*_−1_ are generated by energy transfer from *ω*_0_. (**c**) FWM-2: nondegenerate FWM. Only *ω*_1_ is generated. (**d**) FWM-3: nondegenerate FWM. Only *ω*_−1_ is generated. (**e**) Frequency difference among *Ω*_n_, n = 1 − 3, as a function of intracavity hydrogen pressure. A, B and C correspond to the pressures that satisfy the phase-matching conditions of FWM-1, FWM-2 and FWM-3, respectively.

**Figure 5:**
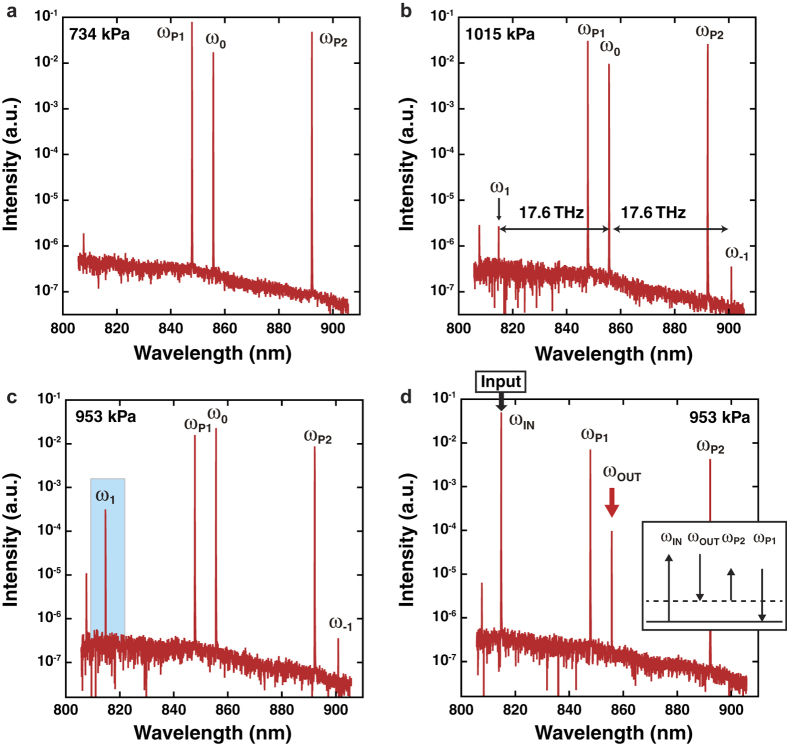
Output beam spectra under three different intracavity hydrogen pressures. (**a**) 734 kPa: no sidebands are observed. (**b**) 1015 kPa: sideband generation as a result of MOM at 17.6 THz is clearly observed. (**c**) 953 kPa: the intensity of the anti-Stokes sideband is substantially enhanced. (**d**) 953 kPa: a beam at a wavelength corresponding to *ω*_0_ is generated for an input beam with a wavelength corresponding to *ω*_1_. The inset shows the energy diagram of FWM required to provide this spectrum.

All frequencies involved in the intracavity MOM process are shown in Fig. 4a. These frequencies include those of the pump beams, *ω*_P1_ and *ω*_P2_, the probe beam, *ω*_0_, and the sidebands, *ω*_1_ and *ω*_−1_. It is noted that three types of FWM shown in Fig. 4b–d actually contribute to the intracavity MOM process. One is DFWM (FWM-1, Fig. 4b) and the other two are nondegenerate FWM mechanisms (FWM-2, Fig. 4c and FWM-3, Fig. 4d). In FWM-1, both sidebands are generated at the expense of *ω*_0_. In contrast, FWM-2 and FWM-3, which involve both *ω*_P1_ and *ω*_P2_, generate only one sideband each: *ω*_1_ or *ω*_−1_, respectively. As mentioned above, the phase-matching conditions for these FWM processes are satisfied when the related longitudinal modes are arranged at equal intervals, e.g. Δ*Ω*_1_ = 0 and Δ*Ω*_2_ = 0 in Fig. 2c for FWM-1. When *Ω*_1_, *Ω*_2_ and *Ω*_3_ are defined by frequency differences between the longitudinal modes related to the intracavity MOM process, as shown in Fig. 4a, the phase-matching conditions then correspond to *Ω*_1_ = *Ω*_2_, *Ω*_2_ = *Ω*_3_ and *Ω*_1_ = *Ω*_3_ for FWM-1, FWM-2 and FWM-3, respectively. Because the group delay of a gaseous medium depends on its concentration, the separation between the longitudinal modes can be controlled by varying the applied pressure in the optical cavity. Figure 4e shows the dependencies of *Ω*_2_ − *Ω*_1_, *Ω*_2_ − *Ω*_3_ and *Ω*_3_ − *Ω*_1_ on intracavity pressure at *ω*_P1_ = 847.6 nm, *ω*_P2_ = 901.1 nm and *ω*_0_ = 855.8 nm; these values were estimated from the designed group delay value of the cavity mirrors and the reported dispersion of gaseous hydrogen^16^. At points A, B and C, the frequency difference is equal to zero, which means that the phase-matching conditions for FWM-1, FWM-2 and FWM-3, respectively, are satisfied at each hydrogen pressure condition. Figure 5a shows the spectrum of the output beam observed when *ω*_P1_ (847.6 nm) and *ω*_0_ (855.8 nm) are coupled to the optical cavity when it is filled with hydrogen at 734 kPa. A relatively strong signal at 891.9 nm (*ω*_P2_) and a weak signal at 807.4 nm are observed. The strong *ω*_P2_ signal is generated through SRS, where the phase-matching condition is automatically satisfied, whereas the weak signal at 807.4 nm is generated by DFWM involving *ω*_P1_ and *ω*_P2_, where the phase-matching condition is not satisfied. Notably, no sidebands (*ω*_1_ and *ω*_−1_) arising from MOM are observed in Fig. 5a. This is apparently because the phase-matching condition for MOM is not satisfied at this hydrogen pressure, although the molecular coherence of the hydrogen molecules in the optical cavity is excited sufficiently by *ω*_P1_ and *ω*_P2_.

### Generation of the sidebands

We find that the output beam spectrum includes both sidebands, *ω*_1_ and *ω*_−1_ at an intracavity hydrogen pressure of 1015 kPa, which is in the vicinity of point A in Fig. 4e, where the FWM-1 phase-matching condition is expected to be satisfied. The difference between the measured pressure (1015 kPa) and the predicted one in Fig. 4e might be caused by a thermal effect associated with the inelastic SRS process. Peaks separated by the hydrogen rotational frequency (17.6 THz) from *ω*_0_ on the longer and shorter wavelength sides are clearly observed at 814.9 nm (*ω*_1_) and 901.1 nm (*ω*_−1_) in Fig. 5b. The ratios of the intensities of the sideband to that of the probe beam (i.e., 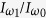 and 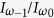) are both 1.4 × 10^−4^. The following three reasons indicate that these peaks are the sidebands that originated from MOM. (1) No sidebands were observed when the pump beam was blocked and the probe beam alone was coupled to the hydrogen-filled optical cavity at a power below the SRS threshold. This suggests that the molecular coherence excited by the pump beam contributes to sideband generation. (2) Strong anti-Stokes emission at *ω*_1_ was observed at hydrogen pressure of 1015 kPa, when the pump beam was blocked and the probe beam power was above the SRS threshold. In this case, *ω*_−1_ is generated through intracavity SRS, and *ω*_1_ arises from DFWM involving *ω*_0_ and *ω*_−1_, even without the pump beams. The phase mismatch in this DFWM process is expressed by Δ*k* = 2*k*_0_ − *k*_1_ − *k*_−1_, which is exactly the same as that in FWM-1 that provides both sidebands in the MOM process. This also indicates that the cavity longitudinal modes are arranged so that *Ω*_1_ = *Ω*_2_ at an intracavity hydrogen pressure of 1015 kPa. (3) The intensity ratio between the sidebands in Fig. 5b is nearly 1:1. As expected from the symmetry of the DFWM process, which originated from a rotational transition pumped by a linearly polarized beam, the Stokes (*ω*_−1_) and anti-Stokes (*ω*_1_) emissions have identical gain coefficients, leading to sidebands with identical intensities in the phase-matched MOM process when probed by a linearly polarized beam. Points (1)–(3) strongly indicate that *ω*_1_ and *ω*_−1_ in Fig. 5b are generated by MOM based on molecular coherence excited by the pump beam under phase-matched conditions.

### Enhancement of the sidebands

We also find that the intensity of one sideband is significantly enhanced at a different pressure from the point that was observed in the preceding paragraph. Figure 5c shows the spectrum observed when the intracavity hydrogen pressure is reduced from 1015 kPa to 953 kPa, giving 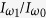 of 7.3 × 10^−3^, which is ~50 times larger than the value observed in Fig. 5b. This enhancement can be explained by the shift from the point at which the phase-matching condition of FWM-1 is satisfied (A in Fig. 4e) to the corresponding point for FWM-2 (B in Fig. 4e), although the hydrogen pressure (953 kPa) deviates slightly from the predicted 930 kPa value. To validate this idea, we observed the output spectrum at the hydrogen pressure at which the sideband enhancement was observed (953 kPa) when the probe beam wavelength was set at 814.9 nm, corresponding to the frequency of *ω*_1_ in Fig. 5c. As a result, an intense signal was observed at the wavelength corresponding to the frequency of *ω*_0_ (855.9 nm) (see Fig. 5d). This emission at 855.8 nm originated from FWM, as shown in an inset of Fig. 5d. This FWM process requires the same phase-matching condition as that for FWM-2 because all frequency components are common in both cases. This clearly indicates that hydrogen pressure of 953 kPa leads to *Ω*_2_ = *Ω*_3_, and both FWM processes satisfy the phase-matching condition at the pressure corresponding to point B in Fig. 4e.

## Discussion

As shown above, the pump beam contribution (*ω*_P1_ and *ω*_P2_) through FWM-2 and FWM-3 is critical to improvement of the MOM process efficiency. While one sideband (*ω*_1_) is substantially enhanced at point B in Fig. 4e, the other sideband (*ω*_−1_) remains at a low level. In fact, the Stokes sideband, *ω*_−1_, shown in Fig. 5c at 901.1 nm is roughly three orders of magnitude smaller than the anti-Stokes sideband. This is apparently because the phase-matching condition required for *ω*_−1_ (FWM-3) generation is not satisfied in this FWM process. To enhance both sidebands, the phase-matching conditions at A, B and C must be satisfied simultaneously at one pressure value, i.e. *Ω*_1_ = *Ω*_2_ = *Ω*_3_. This is achieved by using a constant free spectral range throughout the frequency range, which completely compensates for all orders of dispersion. In reality, however, the optical cavity used in this experiment offers different pressures that are ~100 kPa apart from each other for the three phase-matching points, as shown in Fig. 4e. This is attributed to the higher-order dispersion components included in the total GDD of the hydrogen-filled optical cavity, i.e. even-order terms higher than the fourth order of the total GDD. The cavity mirrors used in this experiment are commercially-available chirped mirrors that have “ripples” in their dispersive properties (see Fig. S1b in the supplementary information) due to Gires–Tournois interference effects^17^. Because the higher-order dispersion contribution from gaseous hydrogen is relatively small, the difference among the pressures that satisfy the phase-matching conditions for FWM-1, FWM-2 and FWM-3 is mainly determined by the high-order dispersion values of the cavity mirrors. This high-order dispersion can be removed by sophisticated design to suppress the GDD ripple^18^ or by destructive cancellation using a pair of chirped mirrors with oscillating GDDs with a *π*-phase difference^19^. Adequate compensation for high-order GDD in a medium-filled optical cavity will allow us to satisfy the phase-matching conditions for FWM-1, FWM-2 and FWM-3 simultaneously, leading to generation of sidebands with higher intensities. It should be noted that perfect dispersion compensation over the entire frequency range of high reflectivity is not necessary because of the discrete nature of the spectrum. This would be helpful to satisfy the phase-matching condition for the highly efficient sideband generation.

**Figure 6:**
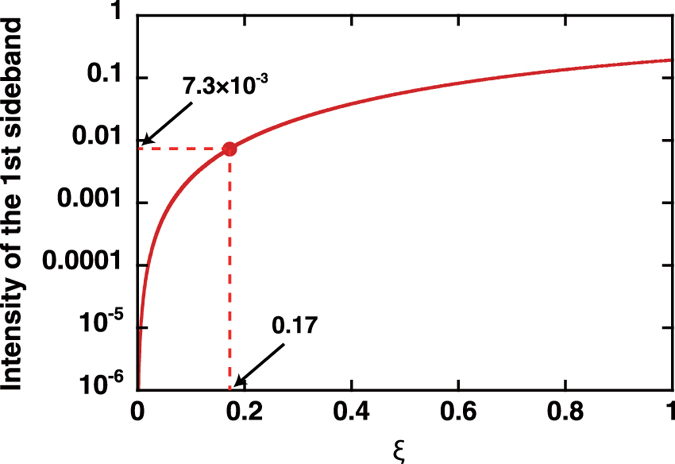
Evolution of the first-order sideband intensity depending on the parameter *ξ*. *ξ* is defined in the main text. The maximum intensity ratio measured in the experiment (7.3 × 10^−3^) corresponds to *ξ* = 0.17 under an assumption of perfect phase-matching.

Another simple approach to enhance the modulation efficiency when using the current optical cavity is to increase the intracavity pump beam power. Under the phase-matching condition described in the previous section, the intensity of the *n*th-order sidebands is expressed by a Bessel function in terms of the parameter *ξ*: 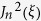. Here, *ξ* is defined using the molecular coherence, *ρ*, as follows^20^,^21^:


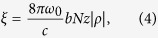


where *b* is a constant related to dipole moments and single-photon detuning from the electronic transition states^22^, *N* is the molecule concentration, and *z* is the interaction distance between the probe beam and the molecular coherence. This equation indicates that the first-order sideband intensity approximately evolves with 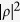 in the range where 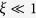. Figure 6 shows the intensity dependence of the first-order sidebands on *ξ*. In the experiment, the largest ratio of 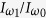 is observed to be 7.3 × 10^−3^, corresponding to *ξ* = 0.17 under the assumption of perfect phase matching. Because *ξ* is proportional to 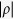, a value of *ρ* that is an order of magnitude larger would lead to a sideband with intensity comparable to *ω*_0_ in the output beam. This is not impossible, because Green *et al.* reported^23^ *ρ* = 3 × 10^−4^, which is one order of magnitude larger than the estimated value in our experiment. Further improvement in the efficiency of MOM allows us to shorten the interaction length between the molecular coherence and the probe beam in the cavity. This reduces the necessity for the strong cavity-enhancement of the probe beam, i.e. the high reflectivity of the cavity mirrors for the probe beam. This will thus open a door for cw-based molecular optical modulators that can be used over wider probe beam wavelength ranges, e.g. from ultraviolet to infrared.

In conclusion, a molecular optical modulator for a cw beam operating at a frequency of more than 10 THz has been demonstrated. This concept is based on ultrafast variation of the polarizability of gaseous molecules that arises from coherent motion. The coherent motion of the molecules is excited using a dual-wavelength cw beam, where the beam intensity is enhanced in a high-finesse optical cavity. A probe beam, which is also enhanced in the optical cavity, undergoes optical modulation as a result of interaction with the excited coherence of the intracavity molecules. The modulation frequency corresponds to the frequency of the molecular motion; in this case, it is a rotational hydrogen molecule motion at 17.6 THz. Careful control of the total GDD of the medium-filled optical cavity plays a critical role in satisfying the phase-matching condition of MOM. Phase-matched MOM was achieved by balancing the negative dispersion of the cavity mirrors with the positive dispersion of the intracavity medium, allowing us to increase the sideband generation efficiency by two orders of magnitude at a pump power level that was two orders of magnitude less than that used in a previous report^9^. The ratio of the intensity of the sidebands to that of the probe beam reached 7.3 × 10^−3^. Unfortunately, at this stage, higher-order dispersion components prevent the fulfilment of all phase-matching conditions. Elimination of the higher-order dispersion problem, which is mainly related to the cavity mirrors, and increased pump powers will improve the modulation performance sufficiently to generate higher order sidebands. The optical modulation of cw radiation in the THz region reported here offers a promising way to push the limits of conventional electro-optic modulation methods, and will lead to applications in broader scientific areas, such as laser engineering, optical communications, precise spectroscopy and other optical science fields. For example, an oscillator producing ultrashort optical pulses and operating at a repetition rate of over 10 THz, i.e. a terahertz mode-locked laser, would be a powerful tool in ultrafast applications.

## Methods

### Estimation of *Ω*_n_

Frequency differences between the longitudinal modes related to the intracavity MOM process are calculated using the sum of free spectral ranges, *δΩ*. For example, the frequency difference between the longitudinal modes related to *ω*_0_ and *ω*_1_ (see Fig. 4a) is given by the following equation:


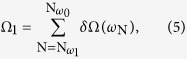


where *ω*_N_ is the frequency of the Nth longitudinal mode, and 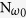 and 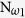 are the numbers of the longitudinal modes responsible for *ω*_0_ and *ω*_1_, respectively. *δΩ*(*ω*) is given by the reciprocal of the group delay for a single round trip of the intracavity beam^12^. The group refractive index of the intracavity medium, *n*_*g*_, is derived from a Sellmeier equation reported in the literature^16^ and the group delay induced by a bounce of the beam from the cavity mirror is obtained by integrating the GDD, as shown in Fig. S1b of the supplementary information.

### Estimation of *ρ*

The concept for creation of high coherence between two states, where the transition between the states is electric-dipole-forbidden, is driven by radiation with multiple far-off-resonance wavelengths and was established by Harris *et al.*^24^. In our research, we use this theoretical model with a minor modification to calculate the molecular coherence between rotational states that are excited using a cavity-enhanced dual-wavelength cw laser. Let us denote the lower and upper values of the two states with driven coherence and the energy difference between these states as *a*, *b* and *ħω*_m_, respectively. These states are excited by cw radiation that includes two components at frequencies of *ω*_p_ and *ω*_s_ (where *ω*_p_ > *ω*_s_), and the difference of these frequencies corresponds to the frequency of molecular motion, i.e. *ω*_p_ − *ω*_s_ = *ω*_m_. Here, the coherence between the states *a*, *b* and *ρ*, can be calculated using the Stark shifts, *Ω*_*aa*_ and *Ω*_*bb*_, and the two-photon Rabi frequency, *Ω*_*ab*_^24^,^25^. When the electronic transitions are far-off-resonant and the population in the upper level is negligible, i.e. the population difference is virtually equal to −1, *ρ* is expressed as follows in the 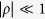 regime when taking the coherence dephasing rate *γ*_*ab*_ into account:


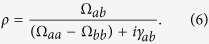


By ignoring the relative Stark shift (*Ω*_*aa*_ − *Ω*_*bb*_ ≈ 0) because of the small *ħω*_m_ value, and using 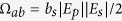, 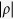 is approximated by:


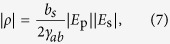


where *b*_*s*_ is a coefficient given by the dipole moments and the single photon detuning from the upper electric states, and 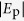 and 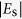 denote the intracavity amplitudes of the pump laser. In the rotational transition of *ortho*-hydrogen (S_0_(1), *ω*_m_/2*π* = 17.6 THz), *γ*_*ab*_/2*π* is 5.1 × 10^8^ Hz at a pressure of 10 atm^26^, and the coefficient *b*_*s*_ can be estimated^27^ to be 4.2 × 10^−8^ Hz/V^2^ from the Raman gain coefficient (0.5 × 10^−11^ m/W)^28^.

## Additional Information

**How to cite this article**: Zaitsu, S.-i. *et al.* Continuous-wave phase-matched molecular optical modulator. *Sci. Rep.* **6**, 20908; doi: 10.1038/srep20908 (2016).

## Supplementary Material

Supplementary Information

## Figures and Tables

**Figure 1:**
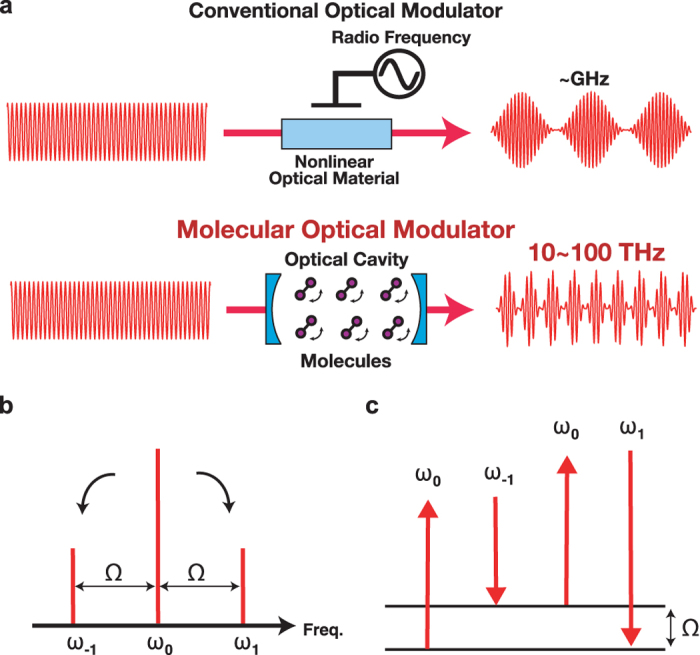
Basic optical modulator concept. (**a**) Top: conventional optical modulator based on the electro-optic effect. Bottom: molecular optical modulator. (**b**) Generation of sidebands arising from optical modulation at a frequency of *Ω. ω*_0_ is a fundamental frequency. *ω*_1_ and *ω*_−1_ are the frequencies of the anti-Stokes and Stokes beams. (**c**) Energy diagram of sideband generation. This corresponds to the process of degenerate four-wave mixing.
