# Supplementary material for: Continuous-wave phase-matched molecular optical modulator
Source: Sci Rep. 2016 Feb 18;6:20908. doi: 10.1038/srep20908 (PMC4757869; doi:10.1038/srep20908)
Supplement: Supplementary Information [file srep20908-s1.pdf]

Continuous-wave phase-matched molecular optical modulator

Shin-ichi Zaitsu[1,2,3], Hirotomo Izaki[1], Takao Tsuchiya[1] & Totaro Imasaka[1,3]

[1]*Department of Applied Chemistry, Graduate School of Engineering, Kyushu University, 744 Motooka, Nishi-ku, Fukuoka 819-0395, Japan*

[2]*PRESTO, Japan Science and Technology Agency (JST), 4-1-8 Honcho, Kawaguchi, Saitama 332-0012, Japan*

[3]*Division of Optoelectronics and Photonics, Center for Future Chemistry, Kyushu University, 744 Motooka, Nishi-ku, Fukuoka 819-0395, Japan*

SUPPLEMENTARY INFORMATION

1 Optical cavity

In this experiment, optical modulation in the THz frequency region is imposed on a continuous-wave (cw) laser (the probe beam) through ultrafast variation of the susceptibility of a gaseous medium, accompanied by coherent molecular motion excited by another cw laser (the pump beam). To reach this goal, a high-finesse optical cavity was used to enhance the power of the pump beam and extend the interaction length between the excited molecular coherence and the probe beam. The cavity mirror requires a broad highly reflective bandwidth that is sufficient to cover the five wavelengths of interest, i.e. the two wavelengths of the pump beam that are separated by the fre-

quency of the molecular motion (modulation frequency: Ω), the probe beam and the first-order sidebands that are separated from the probe beam by Ω on both the higher and lower frequency sides (see Fig. 1b in the main text). Figure S1a shows the transmittance spectrum of the cavity mirrors used in the experiments. When the pump beam wavelengths are set at 847.6 nm and 891.9 nm and the probe beam wavelength is set at 855.8 nm, the first Stokes and anti-Stokes sidebands appear at 814.9 nm and 901.1 nm, respectively. The cavity mirrors have reflectivity of more than 99.96% at all these wavelengths under the assumption that the optical loss is 100 ppm.

We assembled a Fabry–Perot-type optical cavity consisting of a pair of mirrors facing each other with separation of ~80 mm. As described in the main text, one important factor in the realization of MOM is elimination of the total dispersion of the medium-filled optical cavity. The designed group delay dispersion (GDD) spectrum of the cavity mirror provided by the manufacturer (Layertec, GmbH) is shown in Figure S1b. The cavity mirror has a negative GDD of ~-9 fs^2 at the probe beam wavelength. Based on the measured dispersive property of gaseous "normal" hydrogen at room temperature in the near-infrared region that was reported in the literature [1], the positive GDD of 100 kPa hydrogen for one passage through the 8-cm optical cavity can be calculated to be ~1 fs^2. This indicates that the hydrogen molecules should be filled at a pressure of ~900 kPa in the optical cavity to cancel the positive GDD caused by a single passage through the medium in the optical cavity by one bounce on the negatively dispersive cavity mirror.

Figure S2 shows a schematic diagram of the stainless steel chamber used to contain the optical cavity with the gaseous medium used for MOM. The inner diameter of the cylindrical

chamber is 44 mm, and its length is 170 mm. A pair of plano-concave cavity mirrors (diameter: 25.4 mm; radius of curvature: 250 mm) were attached at both ends of a spacer that consisted of a stainless steel tube and a 25-mm-long tubular piezo-actuator. The concave side of the cavity mirror was coated with a multilayer dielectric to provide the broadband highly reflective and negative dispersive properties described in the preceding paragraph. The plano side was coated with an anti-reflective layer to reduce its reflectivity to less than 0.2%. Note that the confocal parameter of this cavity, which was calculated from the radius of curvature of the cavity mirrors (250 mm), is 183 mm, which is much larger than the cavity length (80 mm). This optical cavity (comprising the cylinder equipped with the mirrors at both sides) was installed in the chamber along with a piece of vibration insulator (Sorbothane(R)). Silica substrates with anti-reflection coatings (<0.2%, 670-1070 nm) set on both sides of the chamber were used as windows for introduction of the pump and probe beams and for observation of the output beam. The chamber was also equipped with two ports to accommodate a silicon diaphragm pressure sensor (AP-34, Keyence Corp.) and to provide a feed-through to apply a voltage (<1000 V) to the piezo-actuator and an inlet and outlet for the gaseous media. The maximum travel distance of the piezo-actuator was $\sim$15 μm. The chamber was held in a kinematic mount and the cavity axis was adjusted so that it matched the incident direction of the input beam.

2 Light sources

Two single-frequency tunable Ti:sapphire lasers (line width <100 kHz) were used; one (MBR110, Coherent, Inc.) was used to excite the coherence of the gaseous molecules contained in the optical

cavity (the pump beam), while the other (Solstis, M Squared Lasers, Ltd.) was used as a "probe" beam to measure the optical modulation induced by the coherent molecular motion. We selected these Ti:sapphire lasers as the pump and probe beams because of the availability of broadband dispersion-controlled mirrors in their operating wavelength ranges, their high frequency stability properties, and their wide tunable ranges. While these laser sources are suitable for the first demonstration of a cw molecular optical modulator, semiconductor lasers or fibre lasers operating at other wavelengths will be used if suitable mirrors with well-controlled dispersion properties in the appropriate wavelength regions are available.

3 Coupling of the pump and probe beams to the optical cavity

As a medium for MOM, normal hydrogen ($ortho$: $para$ = 3:1) at room temperature was used in this experiment. The coherence of the hydrogen molecules was created by a beat between beams with two frequencies that were separated by the Raman shift frequency. These beams were generated through intracavity stimulated Raman scattering (SRS) induced by the cavity-enhanced pump beam. The hydrogen molecules were contained in the chamber that contained the Fabry–Perot optical cavity, as described in the previous section. The pump beam was coupled to the optical cavity after it passed through transverse mode-matching optics. The pump beam power just before the chamber was 350 mW. The instantaneous pump beam linewidth is less than the linewidth of a longitudinal mode of the optical cavity, and thus enhances the intracavity power when the frequency of the beam exactly matches one of the longitudinal modes. The finesse that was calculated from the reflectivity of the mirrors is $\sim$10000. The enhanced pump beam intensity

is estimated to be $\sim 4 \times 10^5$ W/cm^2 when the coupling efficiency is taken into account. As described earlier, the bandwidth of the highly-reflective region of the cavity mirror is sufficiently broad to cover the wavelengths of both the pump beam and the Stokes beams. This leads to a dramatic reduction in the threshold for the intracavity SRS [2], allowing us to generate a Stokes beam when the pump beam builds up and exceeds the SRS threshold. Under these experimental conditions, we obtained comparable intensities between the intracavity pump beam and the Stokes beam at the maximum coupling efficiency. Please see the main text for the estimated magnitude of the molecular coherence through the intracavity SRS process.

The probe beam was passed through transverse-mode matching optics and coupled to the optical cavity along with the pump beam. The probe beam power just before the chamber was 60 mW. The probe beam had linear polarization in the direction perpendicular to that of the pump beam, and was spatially combined with the probe beam using a dichroic mirror. The instantaneous linewidth of the probe beam was sufficiently narrow to enable it to be resonant with a cavity longitudinal mode; the pump beam was resonant with a different mode. The detailed protocols required to maintain and stabilize the resonant conditions for both the pump and probe beams are described in a subsequent section. Throughout the experiments, the wavelengths of the pump and probe beams were fixed at 847.6 nm and 855.6 nm, respectively, and the changes in the output spectrum were measured by fine-tuning of the intracavity hydrogen pressure. A spectral analyser (721-A, Bristol Instruments, Inc.) was used to record the output beam spectrum.

4 Cavity stabilization

To maintain the resonance conditions for two beams that were simultaneously coupled to different longitudinal modes of a single optical cavity, two independent systems based on a Pound–Drever–Hall (PDH) scheme [3] were used in this experiment to stabilize the optical cavity. The pump and probe beams were modulated by an optical phase modulator (4061, New Focus, Inc.) at two frequencies (*ca.* 12 MHz) that were slightly different to each other. The probe beam polarization was adjusted to be orthogonal to that of the probe beam (pump: p-polarization; probe: s-polarization), and the beams were overlapped using a dichroic mirror. The designed values of transmittance for the pump beam and reflectance for the probe beam were 98% and 64%, respectively. The quarter-wave plate that is generally used in standard PDH methods was not installed in the system to enable use of a linearly polarized probe beam. The beams that were reflected from the optical cavity included information on the deviation from the resonant condition, and were measured by photodetectors; the signals for the pump and probe beams were detected using the transmitted and reflected beams from the dichroic mirror, respectively. The detected signals were then demodulated using two lock-in amplifiers (SR844, Stanford Research Systems, Inc.) to obtain the required error signals. After they were passed through a servo device (LB1005, New Focus, Inc.), the pump beam signal was fed back to the piezo-actuator, which was equipped with one of the cavity mirrors, and the signal for the probe beam was fed back to a reference cavity in the Ti:sapphire laser to maintain the resonance condition. The cavity length was controlled manually to match the pump frequency with a cavity longitudinal mode before closing the feedback loop to lock the cavity length to the pump beam frequency. After the pump beam locking process, the probe beam fre-

quency was finely tuned to match it with a longitudinal mode and the feedback loop for the probe beam was then closed to lock the frequency. A large part of the difficulty in cavity stabilization for the pump beam was removed by a thermal effect caused by Stokes beam generation [4]. However, the reverse of the locking procedure, i.e. from probe beam locking $\rightarrow$ pump beam locking, caused a break in the lock for the probe beam, which was possibly caused by the thermal effect.

References

1. Herring, G. C., Dyer, M. J. & Bischel, W. K. Temperature and density dependence of the linewidths and line shifts of the rotational Raman lines in N_2 and H_2. *Phys. Rev. A* **34,** 1944-1951 (1986).

2. Brasseur, J. K., Repasky, K. S. & Carlsten J. L. Continuous-wave Raman laser in H_2. *Opt. Let.* **23,** 367-369 (1998).

3. Drever, R. W. P. *et al.* Laser phase and frequency stabilization using an optical resonator. *Appl. Phys. B* **31,** 97-105 (1983).

4. Zaitsu, S. & Imasaka, T. Continuous-wave multifrequency laser emission generated through stimulated Raman scattering and four-wave Raman mixing in an optical cavity. *IEEE J. Quantum Electron.* **47,** 1129-1135 (2011).

Figure 1 Characteristics of cavity mirror used in this experiment. a, Measured transmission spectrum. b, Designed group delay dispersion.

Figure 2 Schematic of gas-filled chamber containing the optical cavity.

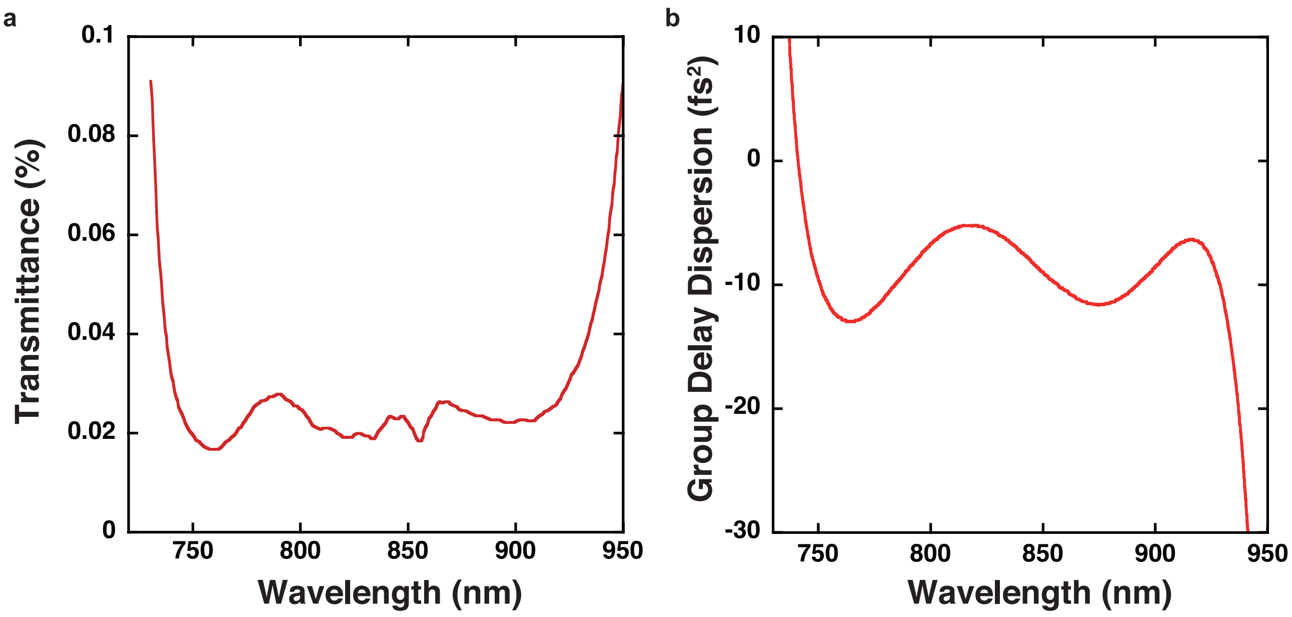
a
b
Transmittance (%)
0.1
0.08
0.06
0.04
0.02
0
750
800
850
900
950
Wavelength (nm)
Group Delay Dispersion (fs^2)
10
0
-10
-20
-30
750
800
850
900
950
Wavelength (nm)

Figure. S1

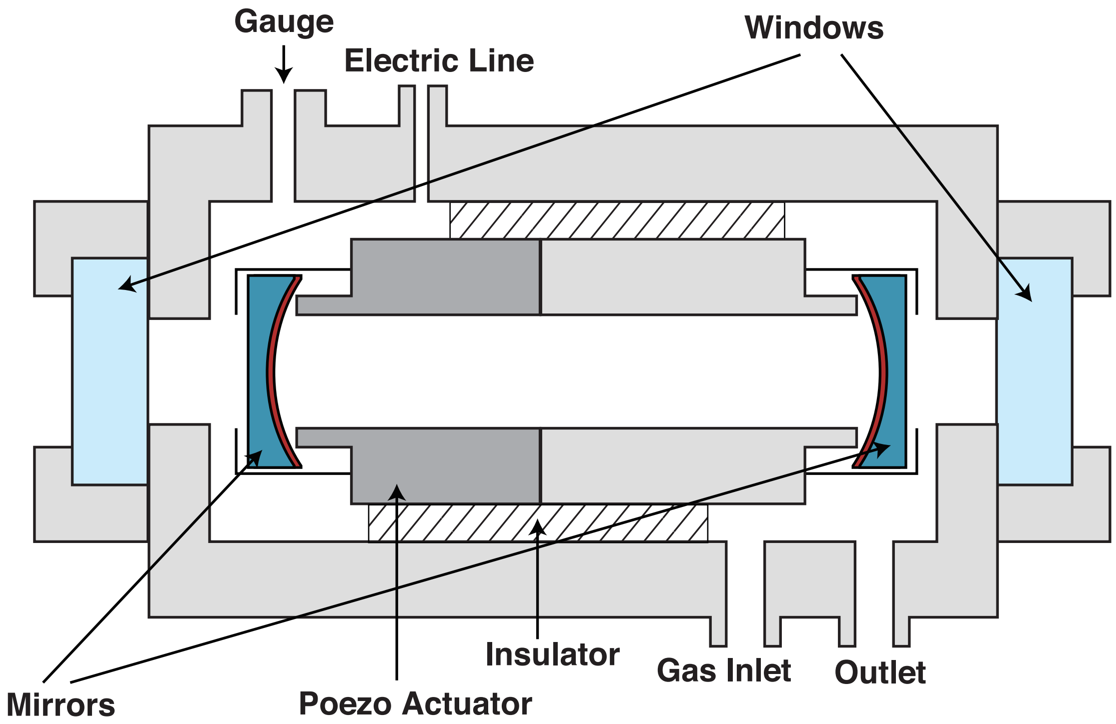
Gauge
Electric Line
Windows
Insulator
Gas Inlet
Outlet
Mirrors
Poezo Actuator

Figure. S2
